# Supplementary material for: The gap between desired and expected fertility among women in Iran: A case study of Tehran city
Source: PLoS One. 2021 Sep 14;16(9):e0257128. doi: 10.1371/journal.pone.0257128 (PMC8439469; doi:10.1371/journal.pone.0257128)
Supplement: Attachment — Submitted filename: Response to Reviewers comments 9 June.docx [file pone.0257128.s002.docx]

**Responses to reviewers’ comments**

**Title: The gap between desired and expected fertility among women in Iran: A case study of Tehran city
PLOS ONE**

**Reviewer’s comments**

Reviewer #1:  Although authors have tried to address all comments, two comments have not been appropriately addressed. Against the comment 3 and 4, authors did not give suitable responses and did not adequately make necessary changes in the manuscript. Authors are advised to analyse two things and include them in the paper. The level of mismatch of desired fertility and actual fertility by parity and sex-composition (using an additional definition). Thus, the authors may estimate the mean CEB, mean additionally intended number of children, mean expected CEB, mean desired family size, and gaps by the number of children delivered (parity) such as 1, 2, and 3+. Similarly, the same estimates may be produced by the women who have at least one son and who don’t have a son.

Author’s response: The analysis suggested by Reviewer #1 has been added in the form of. two tables - Table 4 and Table 7 and their interpretation, to address the level of mismatch of desired fertility and actual fertility by women’s parity and sex-composition of their children. There was no further comment from Reviewer#2.
